# Development and evaluation of the HRSD-D, an image-based digital measure of the Hamilton rating scale for depression

**DOI:** 10.1038/s41598-022-18434-y

**Published:** 2022-08-22

**Authors:** Adi Berko, Avigail Bar-Sella, Hadar Fisher, Michael Sobolev, J. P. Pollak, Sigal Zilcha-Mano

**Affiliations:** 1 The Department of Psychology, University of Haifa, Mount Carmel, 31905 Haifa, Israel; 2 Feinstein Institutes for Medical Research, Northwell Health, New York, USA; 3 Cornell Tech, Cornell University, New York, USA

**Keywords:** Health care, Drug therapy

## Abstract

The Hamilton rating scale for depression (HRSD) is considered the gold standard for the assessment of major depressive disorder. Nevertheless, it has drawbacks such as reliance on retrospective reports and a relatively long administration time. Using a combination of an experience sampling method with mobile health technology, the present study aimed at developing and conducting initial validation of HRSD-D, the first digital image-based assessment of the HRSD. Fifty-three well-trained HRSD interviewers selected the most representative image for each item from an initial sample of images. Based on their responses, we developed the prototype of HRSD-D in two versions: trait-like (HRSD-DT) and state-like (HRSD-DS). HRSD-DT collects one-time reports on general tendencies to experience depressive symptoms; HRSD-DS collects daily reports on the experience of symptoms. Using a total of 1933 responses collected in a preclinical sample (*N* = 86), we evaluated the validity and feasibility of HRSD-D, based on participant reports of HRSD-DT at baseline, and 28 consecutive daily reports of HRSD-DS, using smartphone devices. HRSD-D showed good convergent validity with respect to the original HRSD, as evident in high correlations between HRSD-DS and HRSD (up to Bstd = 0.80). Our combined qualitative and quantitative analyses indicate that HRSD-D captured both dynamic and stable features of symptomatology, in a user-friendly monitoring process. HRSD-D is a promising tool for the assessment of trait and state depression and contributes to the use of mobile technologies in mental health research and practice.

## Introduction

Major depressive disorder (MDD) is the leading cause of disability worldwide, with more than 260 million people affected^1^. The burden of MDD is on the rise globally, making the improvement of treatments a high priority (World Health Organization^1^). Although treatments are overall effective in reducing symptoms, less than half the patients display full remission^2,3^. The reason may lie in problems of symptom monitoring, as insensitive monitoring may impede the identification of subtle differences between individuals and between patterns of symptomatology.

Available monitoring methods can be divided into two main categories: self-report questionnaires (such as the Beck Depression Inventory, BDI, Beck et al.^4^) and semi-structured interviews (such as the Montgomery-Asberg Depression Rating Scale^5^). One of the common tools for MDD symptom monitoring is the Hamilton Rating Scale for Depression (HRSD), a semi-structured interview, containing 17 items assessing the patient's symptoms in the preceding week^6^. This scale is the most commonly used tool for evaluating MDD symptoms in psychotherapy and psychiatric research, and is considered a gold standard^7–9^. Despite its extensive use, the HRSD has been criticized for the unequal contribution of the items to the global score, due to their different scaling, and for the poor inter-rater and retest reliability displayed by many of its items^10^.

In addition to these psychometric drawbacks, the HRSD has some conceptual drawbacks, which also characterize other traditional MDD monitoring tools. First, HRSD has low ecological validity, as the interview takes place in clinical or lab settings, while asking patients to retrospectively report on their everyday experiences^11^. As the memory of depressed individuals is negatively biased^12,13^, relying on their retrospective reports is problematic. Second, HRSD consumes a considerable amount of time and resources, as patients are asked to attend the interview weekly, and each interview may take 30 min to complete^7^. This procedure, which relies heavily on the patient's cooperation, might be very challenging, given the poor motivation that usually characterizes MDD^14,15^. Third, HRSD is usually based on a weekly assessment of symptoms^8^, and occasionally, assessment points are even more distant in time (e.g., Moran & Mohr, 2005). This weekly (or even monthly) monitoring is partial, as a great deal of information regarding symptomatology patterns is missing^16^. Finally, HRSD fails to distinguish between trait-like and state-like components of symptomatology; that is, between stable general features of the individual's symptomatology and the dynamic daily manifestation of symptoms. Distinguishing between them is essential for the understanding of mechanisms of change in treatments and for the personalization of treatments^17,18^. These drawbacks of the HRSD create a need for additional, complementary tools.

A potential solution for the low ecological validity of the HRSD can be found in the well-supported experience sampling method (ESM) and ecological momentary assessment (EMA). ESM/EMA have already been used in research into mental conditions, including depression^19–21^. In ESM/EMA studies, people react to repeated assessments and report their experiences while functioning in their everyday settings. This real-time assessment reduces memory biases^22,23^. In addition, the frequent assessment of the ESM/EMA seems to better capture the dynamic pattern of symptoms, addressing another drawback of the original tool^24^. As for efficiency, the integration of smartphones into ESM/EMA research has taken the field forward, providing the opportunity to create new assessment tools which are considerably more efficient and less demanding for participants than the traditional ones^25,26^.

The use of smartphones in physical and mental medical research, especially for remote monitoring, is becoming increasingly common^27,28^. To ensure high efficiency and keep the monitoring process user-friendly for participants, some researchers have used non-verbal (e.g., image-based) formats (e.g., Arthritis symptom monitoring^29^). Non-verbal digital tools have also been used to capture complicated concepts, such as emotional states and mental symptoms, in clinical and non-clinical samples^30–32^. Non-verbal digital ESM/EMA assessment tools enable a quick and intuitive response, and thus, are considered to be better suited for populations with poor motivation, such as MDD patients^31,33^.

In light of these significant advantages, ESM/EMA in their technological form (e.g., smartphones), have spread in psychotherapy and psychiatric research, as a means of monitoring the progress of treatment^34^. Yet, this trend is still in its infancy, with not enough evidence-based tools available^34–37^. Even fewer attempts have been made to develop digital ESM/EMA versions of existing MDD monitoring tools^36^. Two such studies sought to develop digital versions (apps) of the Center for Epidemiologic Studies Depression Scale-Revised (CESD-R) and of the Patient Health Questionnaire-9 (PHQ-9^38,39^). Both studies displayed promising results in terms of validity and adherence rates. However, neither of them used a non-verbal format, which is better suited for the engagement of MDD patients^31,33^. Furthermore, neither of the studies distinguished between trait-like and state-like aspects; that is, between a baseline report on the individual's general tendencies to experience MDD symptoms, and repeated reports on the daily experience of symptoms. In addition, to our knowledge, no previous effort has been made to develop a digital ESM/EMA version of the HRSD, despite being the most commonly used MDD scale in randomized controlled trial research^7,8^. Therefore, to the best of our knowledge, our project is the first attempt to create an ESM/EMA digital version of the gold standard HRSD, in order to monitor MDD symptoms during treatment, while addressing the mentioned drawbacks of the original tool.

### The present study

The aim of the present study was to develop a digital tool for monitoring MDD symptoms during treatment. The development of HRSD-D, a digital image-based version of the gold standard HRSD, aims to address four drawbacks of the existing tool: low ecological validity, low efficiency, missing information due to long intervals between assessments and lack of discrimination between state-like and trait-like aspects of symptomatology. HRSD-D collects daily real-time reports on MDD symptoms by smartphone and in everyday settings, unlike the original HRSD, which is a retrospective report based on a weekly interview in a clinical or lab setting. To improve efficiency and make HRSD-D as user-friendly as possible, we used images to report symptoms. This approach of using non-verbal content was found efficient and effective in assessing mental health symptoms and in differentiating between emotional states in clinical and non-clinical samples^30–33,40–42^. The current study constitutes the first phase of the HRSD-D development program, and thus a prototype version was used for validation on a preclinical sample. The study focused on three main aims: (a) development of HRSD-D (two versions: HRSD-DS, state-like, and HRSD-DT, trait-like) by the selection of the images to be included; (b) validation of the two versions of HRSD-D; (c) assessment of the feasibility of HRSD-DS by examining its ability to capture the dynamic features of MDD manifestations occurring in parallel with significant stable features of symptomatology, and (d) replication in an independent sample.

## General method

We developed and evaluated HRSD-D in three stages. In stage 1, we created a pool of items consisting of three potential images for each original HRSD item and asked well-trained HRSD interviewers to select the most representative image for each item. Based on the results of stage 1, we developed the prototypes of HRSD-DS and HRSD-DT. To disentangle the two components, we used two versions of the same construct with different instructions^17^ (e.g., STAI^43^). In stage 2, we evaluated HRSD-D on a preclinical sample and tested its validity and feasibility using qualitative and quantitative approaches. In stage 3 we replicated the feasibility findings of stage 2, using an independent sample.

## Stage 1: development of HRSD-D

We aimed to find a single representative image for each original HRSD item, to be included in HRSD-D. We focused on the first 17 items of HRSD (HRSD-17), a commonly used version in psychotherapy and psychiatric research^44^.

### Method

The preparatory process for stage 1 included consultations with a focus group of well-trained HRSD interviewers. Three items were discussed (retardation, agitation, and insight) because their evaluation is based on the interviewer's observation. The focus group led to the exclusion of two items, retardation and insight, which are based on the interviewer's observation, and the inclusion of one item, agitation, which is based to a larger degree on self-report. This process resulted in the inclusion of 15 items in HRSD-D. In addition, we added short titles to the images to make them easier to understand. Stage 1 included the following two phases: (a) finding three potential images for each HRSD item, and (b) selecting the most representative image for each item.

In the first phase, we looked for three potential images for each of the 15 HRSD items included in HRSD-D. To this end, we used "Thinkstock" online images (since then, moved to iStock: https://www.istockphoto.com/). In the second phase, we created an online survey, using "Google Forms," asking the respondents (*n* = 53) to choose one out of the three potential images for each item. The survey included animations and human images of different genders. The respondents were licensed clinical psychologists, and undergraduate and graduate students in psychology, working directly with individuals with MDD. Every HRSD item was presented in a separated block of the survey, which included the three potential images, with a title above each, and the text of the original item below them, as well as the question: "Which image do you find to be the most representative of this item?" After completing the 15 blocks of the survey, respondents were asked how well they thought the images captured the items overall. Answers were provided on a 1–5 scale. Respondents were also asked whether they thought the short titles were essential for understanding the items. At the end of the survey, respondents had the opportunity to comment in an open format. The procedure for image selection and the selection of the sample size of respondents were in accordance with previous studies examining evidence-based digital image-based tools for mental health^31,45^.

### Results

Respondents' answers indicated that, overall, the images successfully captured the idea of the original items (M = 3.96, SD = 0.8), and 85% confirmed that the short titles were essential. For 13 out of the 15 items, a single image was selected to be included in HRSD-D, based on the majority of votes. For the remaining two items (insomnia—early in the morning and hypochondriasis) the respondents' open-format feedback indicated that none of the potential images were good enough to represent them. For these two items, a second round of the survey was conducted, using a small sample of respondents who were well-trained in HRSD administration (*n* = 10). Eventually, 15 images were selected to be included in HRSD-D.

## Stage 2: evaluation of HRSD-D

Based on the results from stage 1, we used the Qualtrics software to construct the prototypes of the two versions of HRSD-D: HRSD-DS and HRSD-DT. At this stage, we evaluated the validity and feasibility of HRSD-D on a preclinical sample, using both qualitative and quantitative approaches. We tested convergent validity against the original HRSD. To test the feasibility of HRSD-DS, we followed Wright and Simms’s^46^ analyses of the daily dynamics of personality disorders. We examined the ability of HRSD-D to capture the dynamic nature of MDD symptom manifestations, as well as significant stable features of symptomatology (levels of symptoms and of fluctuations). The feasibility of HRSD-D was assessed also based on qualitative feedback from the participants and by calculating adherence rates across the month.

### Method

#### Participants

Fifty participants reported on their history of depression or similar mood affective disorders, such as anxiety or dysthymia. Recruitment was based on non-probabilistic convenience sampling and snowball sampling methods, which are common in pilot studies^47,48^, including participation of first-year undergraduate students (see Table 1 for the demographic characteristics of the sample, and Table S6 in the online supplement for clinical characteristics).

**Table 1:** Demographic characteristics of the sample.

	*n*	%
**Gender**		
Female	24	48
Male	26	52
**Marital status**		
Single	31	62
Married/partnered	17	34
Divorced/widowed	2	8
**Highest educational level**		
High school/some college	36	72
Graduate college	10	20
Graduate degree	2	8

*N* = 50. Participants were on average 29.12 years old (*SD* = 11.44).

#### Procedure

Potential participants were asked to attend an introductory session, in which the procedure was explained in great detail. The procedure was approved by the Ethics Committee of the University of Haifa, and the experiment was performed in accordance with relevant guidelines and regulations. All participants signed an informed consent form. In the introductory session, participants completed the HRSD. Next, using their smartphones, they completed the two digital questionnaires (HRSD-DT and HRSD-DS). Participants were then instructed to complete the HRSD-DS questionnaire every day for the following 28 consecutive days, roughly at the same time of day. Every day, a link was sent to the participants’ smartphones by SMS at the same time of day, in accordance with the participants' preference and their awakening patterns. Once a week, participants underwent the original HRSD interview, on the same day of the week. The final session included a semi-structured interview regarding the user experience. Goodwin et al.^49^ emphasized the important role of service users (patients) in the evaluation of mental health apps. Transforming HRSD-D into an app is one of the possible future development paths for this tool.

#### Measures

##### Hamilton rating scale for depression (HRSD-17)^6^

A 17-item clinically administered measure assessing the severity of depression. The final score, ranging from 0 to 52, is calculated by summing the 17 items. Higher scores indicate more severe depression. Interviews were conducted by one of the authors, who is highly trained and experienced in the administration and coding of the HRSD. The interviewer was blind to the HRSD-D reports of the participants until the end of the study period.

##### HRSD-D state-like (HRSD-DS)

A daily digital ESM assessment tool of MDD symptoms. HRSD-DS is a digital image-based version of the HRSD (HRSD-17), consisting of a single image for each of the original HRSD items (excluding insight and retardation). Each time the participants start the questionnaire, a screen with instructions is displayed, asking them to recall the preceding day, including sleep quality, activities, and emotional states. Next, 15 images are presented vertically, with a short title for each, and the question "How well does this image represent me in the past 24 h?" below the image. Participants are asked to rate every image on a scale of 1 (not at all) to 5 (very much). A scale is presented below the question and participants answer by pressing the number. HRSD-DS calculates a daily score by summing up all the 15 items (ranging from 15 to 75), with higher scores representing higher severity of MDD symptoms.

##### HRSD-D trait-like (HRSD-DT)

The trait-like version of HRSD-D is intended to produce a baseline measure. The questionnaire is identical to HRSD-DS, with one difference: it asks the participants to report their general tendency to experience MDD symptoms. Participants are therefore asked to recall their emotional tendencies during their adulthood. For each image, the question is formulated as follows: "How well does this image represent me in general?".

##### Qualitative interview

Semi-structured interview, asking participants about their experience with HRSD-DS (the version that was used repeatedly). Interviews took place at the last session and were conducted by the author. The interview focused on three main issues: (a) strengths of HRSD-DS, (b) weaknesses of HRSD-DS, and (c) key principles in developing the final tool or app. The guiding questions were as follows: How was the HRSD-DS experience? Did you have any problems with the tool? Did you find any weaknesses in the tool? What did you like about completing the questionnaire? What do you think are the key principles that need to be followed in developing the final tool? All interviews were recorded and transcribed.

#### Statistical Analyses

##### Validity of HRSD-D

The data were hierarchically nested, with assessments nested within individuals. To account for the resulting non-independence of assessments, and to prevent inflation of effects, we added the individual as a random effect to the analyses, using the SAS PROC MIXED procedure for multilevel modeling (MLM)^50^. To test the validity of HRSD-DS, we investigated whether the daily HRSD-DS scores in a given week tended to covary with the weekly HRSD interview score for the same week. We conducted a series of multilevel models (MLM) to compute the correlations between the one-week averages of HRSD-DS scores and weekly HRSD scores. As for HRSD-DT, it is supposed to reflect general tendencies, and thus we examined its validity against the monthly averages. We tested the correlation between HRSD-DT scores and the average of the HRSD interviews conducted during the month. We also examined the correlation between HRSD-DT scores and the monthly average of the daily HRSD-DS scores. Post-hoc power analyses, supporting the ability of the sample size to produce accurate estimates and item-wise analysis ensuring the structural equivalence of the HRSD-D, are available in the online supplement.

##### Daily fluctuations in MDD symptoms

To test the ability of HRSD-DS to capture the daily fluctuations in MDD symptoms, we first calculated proportions of item endorsement and descriptive statistics for each HRSD-DS item. Next, we examined the proportion of total variance in each item attributable to individual differences (between-persons variability) in contrast to daily fluctuations (within-person variability). To isolate the variance in daily expressions of MDD attributable to individual differences, we calculated the intraclass correlation coefficient (ICC) from unconditional MLMs, with HRSD-DS items as the outcomes. This measure can be interpreted as the proportion of variance at the between-persons level. Within-person variance is then calculated as 1.00—ICC.

##### Stability of symptom levels and fluctuations

To test the ability of HRSD-DS to capture the stable features of individual symptomatology, and ascertain whether individuals maintain their relative position to each other in their level and variance^46^, we investigated the stability of individual differences in average levels of symptoms and average levels of fluctuations. We divided individual time series into quarters (weeks 1, 2, 3, and 4) and calculated individual means (iMs) and individual standard deviation (iSDs) for each quarter. We then correlated the resulting iM and iSD scores for each quarter. This autocorrelation represents the degree of similarity between a given quarter and a lagged version of itself over successive quarters.

##### Predicting state items based on corresponding trait items

Finally, we sought to investigate the relation between the trait-like and state-like scores. To this end, we examined whether HRSD-DT scores predict individual differences in HRSD-DS scores, using MLMs. In these models, HRSD-DS items served as the Level 1 outcomes and were regressed on HRSD-DT items adjusted for gender and age at Level 2.

### Results

#### Validity of HRSD-D

The available data collected suggest that mean administration time of daily reports with HRSD-DS was 94.32 s (SD = 94.44), and adherence rate for HRSD-DS was 96.29% over the 28-day study period. The mean HRSD-D and HRSD scores as well as their mean item scores are presented in Table 2, which shows that HRSD-DS items vary between patients and time measurement, as indicated by their SD. Results of the multilevel modeling analyses relating the one-week average of HRSD-DS scores to weekly HRSD scores are presented in Table 3. Over the four-week study period, the one-week average of daily HRSD-DS scores correlated significantly and positively with the HRSD score obtained at the interview conducted the same week. The correlation between the two measures and the proportion of shared variance was high, ranging from 50 to 62%. Additionally, HRSD-DT scores correlated positively and significantly with the monthly average of the HRSD scores (r = 0.66, *p* < 0.001), and with the monthly average of HRSD-DS scores (r = 0.82, *p* < 0.001).

**Table 2:** Means scores and SD of the HRSD-DT HRSD-DS and HRSD.

	HRSD-DS	HRSD-DT	HRSD
	Mean (SD)	Mean (SD)	Mean (SD)
Depressed mood	2.21 (1.20)	2.78 (1.20)	1.16 (0.94)
Feelings of guilt	2.20 (1.17)	2.78 (1.35)	1.11 (0.94)
Suicidal thoughts	1.12 (0.49)	1.22 (0.52)	0.19 (0.47)
Difficulties falling asleep	2.09 (1.27)	2.42 (1.25)	0.68 (0.77)
Restless sleep	2.03 (1.25)	2.40 (1.25)	0.60 (0.74)
Early spontaneous awakening	1.98 (1.34)	2.36 (1.40)	0.53 (0.71)
Low motivation (work/activities)	2.25 (1.32)	2.91 (1.38)	1.05 (1.02)
Agitation	2.42 (1.17)	2.80 (1.18)	0.59 (0.65)
Anxiety	2.35 (1.20)	2.98 (1.10)	1.28 (0.98)
Somatic symptoms of anxiety	1.93 (1.11)	2.49 (1.37)	0.92 (1.00)
Loss of appetite	1.52 (0.90)	1.69 (1.00)	0.23 (0.47)
Low energy	2.49 (1.41)	3.18 (1.45)	0.89 (0.78)
Low sexual desire	1.71 (1.12)	2.16 (1.26)	0.36 (0.64)
Hypochondriasis	1.68 (1.02)	2.22 (1.18)	0.59 (0.85)
Loss of weight	1.24 (0.59)	1.64 (1.00)	0.10 (0.32)
Total	29.22 (10.09)	36.02 (10.78)	10.26 (6.67)

HRSD-D items scores range between 1 and 5, HRSD items scores range between 0 and 3.

**Table 3:** Correlations between one-week average of HRSD-DS scores and weekly HRSD score across the four weeks of study.

	Week									
	1		2		3		4		All data	
	Beta	R2	Beta	R2	Beta	R2	Beta	R2	Beta	R2
Weekly HRSD-DS average	***0.76	***.0.58	***0.70	***0.50	***0.80	***0.62	0.77	***.59	***0.77	***0.59

**p < 0.01, ***p < 0.001.

Beta coefficient (standardized) and r square from linear regression predicting HRSD by HRSD-D.

#### Daily fluctuations in MDD symptoms

We examined the proportion of variance in daily HRSD-DS scores attributable to between-persons differences by calculating ICCs from intercept only MLMs. ICCs for HRSD-DS items are shown in Table 4 (note that in Tables 4, 5, 6, 7, 8 the names of the items are listed according to the titles displayed in HRSD-D, not necessarily as they appear in the original HRSD). All ICCs were significant (*p* < 0.001). At the item level, the average ICC was 0.57 (range: 0.25–0.77). This suggests that, on average, approximately 60% of the variance in the daily manifestation of MDD symptoms can be attributed to individual differences, and the remaining 40% to daily fluctuations. At the same time, we found differences depending on the individual item. The items concerning suicidal thoughts, loss of appetite and loss of weight has the lowest ICC indicating that most of the variance in their manifestations was due to daily fluctuations. Feelings of guilt, low motivation, anxiety, somatic symptoms of anxiety, low energy, low sexual desire and hypochondriasis were associated with the largest ICCs, indicating that most of the variance in their manifestations was due to stable individual differences, rather than daily fluctuations. Table 4 also summarizes patterns of endorsement for each HRSD-DS item. The third column of the table shows that the items varied considerably in the proportion of the sample that endorsed them, ranging from 70% and more of the sample that endorsed agitation and anxiety, to only 8% of the sample that endorsed suicidal thoughts, and 17% that endorsed loss of weight.

**Table 4:** Descriptive statistics for endorsement of daily manifestations of MDD symptoms based on HRSD-DS.

Item		Percentage endorsement					
		Ever	Daily				
	Estimated coefficient of reliability, ICC (95% CI)	> 1	1	2	3	4	5
Depressed mood	0.50 (0.39, 0.61)	63	37	27	18	13	5
Feelings of guilt	0.62 (0.52, 0.71)	63	37	27	20	12	4
Suicidal thoughts	0.25 (0.17, 0.36)	8	92	5	2	0	1
Difficulties falling asleep	0.52 (0.41, 0.63)	54	46	22	17	8	7
Restless sleep	0.58 (0.48, 0.68)	50	50	18	17	9	6
Early spontaneous awakening	0.59 (0.48, 0.69)	45	55	17	11	8	9
Low motivation (work/activities)	0.61 (0.50, 0.70)	59	41	21	17	13	8
Agitation	0.57 (0.46, 0.67)	73	27	28	25	15	5
Anxiety	0.61 (0.51, 0.71)	70	30	28	24	11	7
Somatic symptoms of anxiety	0.63 (0.53, 0.72)	50	50	22	17	9	2
Loss of appetite	0.42 (0.32, 0.54)	31	69	17	9	4	1
Low energy	0.68 (0.58, 0.76)	31	34	22	18	14	13
Low sexual desire	0.72 (0.63, 0.80)	36	64	13	14	3	5
Hypochondriasis	0.62 (0.52, 0.72)	38	62	18	13	6	2
Loss of weight	0.45 (0.34, 0.56)	17	83	12	4	1	0
Total	0.77 (0.69, 0.84)	100					

Person-level *N* = 50; daily-level *N* = 952; ICC = intraclass correlation; CI = confidence interval.

**Table 5:** Stability in individual level of symptoms (Mean) over 4 weeks of the assessment period.

Daily item	r 1–2	r 1–3	r 1–4	r 2–3	r 2–4	r 3–4	Mean	Min	Max
	Mean level (iM)								
Depressed mood	0.76	0.80	0.81	0.80	0.74	0.80	0.79	0.74	0.81
Feelings of guilt	0.86	0.71	0.73	0.88	0.86	0.89	0.82	0.71	0.89
Suicidal thoughts	0.50	0.74	0.67	0.57	0.46	0.87	0.64	0.46	0.87
Difficulties falling asleep	0.81	0.77	0.61	0.80	0.75	0.92	0.78	0.61	0.92
Restless sleep	0.79	0.83	0.74	0.79	0.84	0.84	0.81	0.74	0.84
Early spontaneous awakening	0.75	0.75	0.71	0.81	0.78	0.83	0.77	0.71	0.83
Low motivation (work/activities)	0.89	0.81	0.91	0.86	0.92	0.82	0.87	0.81	0.92
Agitation	0.69	0.75	0.78	0.71	0.63	0.85	0.74	0.63	0.85
Anxiety	0.79	0.88	0.77	0.77	0.68	0.87	0.79	0.68	0.88
Somatic symptoms of anxiety	0.88	0.92	0.75	0.89	0.79	0.87	0.85	0.75	0.92
Loss of appetite	0.69	0.73	0.70	0.74	0.54	0.77	0.70	0.54	0.77
Low energy	0.91	0.93	0.94	0.90	0.94	0.96	0.93	0.90	0.96
Low sexual desire	0.62	0.65	0.63	0.93	0.93	0.94	0.78	0.62	0.94
Hypochondriasis	0.81	0.67	0.74	0.93	0.83	0.73	0.79	0.67	0.93
Loss of weight	0.66	0.70	0.67	0.76	0.67	0.79	0.71	0.66	0.79
Items r's mean	0.76	0.78	0.74	0.81	0.76	0.85			
Items r's Min	0.50	0.65	0.61	0.57	0.46	0.73			
Items r's Max	0.91	0.93	0.94	0.93	0.94	0.96			

*N* = 50 All correlations greater than 0.36 are significant at *p* < 0.05.

**Table 6:** Stability in individual levels of fluctuations (SD) over 4 weeks of the assessment period.

	Variability (iSD)						Mean
Depressed mood	0.32	0.50	0.10	0.42	0.20	− 0.20	0.22
Feelings of guilt	0.53	0.58	0.48	0.59	0.40	0.56	0.52
Suicidal thoughts	0.63	0.69	0.54	0.51	0.36	0.41	0.52
Difficulties falling asleep	0.51	0.49	0.39	0.29	0.36	0.48	0.42
Restless sleep	0.54	0.20	0.26	0.50	0.47	0.46	0.41
Early spontaneous awakening	0.58	0.55	0.44	0.48	0.40	0.72	0.53
Low motivation (work/activities)	0.65	0.66	0.33	0.51	0.32	0.10	0.43
Agitation	0.07	0.47	0.42	0.29	0.06	0.67	0.33
Anxiety	0.31	0.49	0.48	0.55	0.27	0.59	0.45
Somatic symptoms of anxiety	0.24	0.47	0.37	0.29	0.41	0.45	0.37
Loss of appetite	0.61	0.52	0.56	0.65	0.54	0.71	0.60
Low energy	0.58	0.51	0.34	0.42	0.21	0.23	0.38
Low sexual desire	0.23	0.59	0.35	0.58	0.78	0.53	0.51
Hypochondriasis	0.10	0.23	0.23	0.74	0.31	0.33	0.32
Loss of weight	0.53	0.69	0.63	0.46	0.60	0.65	0.59
Mean r's items	0.43	0.51	0.39	0.49	0.38	0.45	0.44

*N* = 50 All correlations greater than 0.36 are significant at *p* < 0.05.

**Table 7:** Predicting individual differences in rates of daily state items from their corresponding baseline traits.

Item	Estimate	SE	Partial η^2^	*p*
Depressed mood	0.50	0.04	0.15	< .0001
Feelings of guilt	0.33	0.03	0.12	< .0001
Suicidal thoughts	0.30	0.03	0.11	< .0001
Difficulties falling asleep	0.52	0.03	0.21	< .0001
Restless sleep	0.43	0.04	0.13	< .0001
Early spontaneous awakening	0.50	0.04	0.17	< .0001
Low motivation (work/activities)	0.63	0.04	0.25	< .0001
Agitation	0.43	0.03	0.14	< .0001
Anxiety	0.62	0.04	0.22	< .0001
Somatic symptoms of anxiety	0.40	0.03	0.21	< .0001
Loss of appetite	0.33	0.04	0.07	< .0001
Low energy	0.64	0.02	0.42	< .0001
Low sexual desire	0.30	0.03	0.11	< .0001
Hypochondriasis	0.36	0.03	0.11	< .0001
Loss of weight	0.14	0.02	0.06	< .0001
Total	0.54	0.03	0.27	< .0001

Person-level *N* = 50; daily-level *N* = 952. All models were estimated controlling for gender and age.

partial η^2^: represent an estimate of how much variance in the state item is accounted for by the trait item. According to Cohen (1988) 0.01 partial η^2^ effect considered as small, 0.06 considered as medium and 0.14 considered as large.

**Table 8:** Summary of themes and sample responses extracted from participants' feedback.

Theme	Sample responses
**Strengths of HRSD-D**	
Functionality	Easy; Fast; Very simple; Intuitive Suitable scale
Image-based format	They [the images] make it [the process of reporting] faster Colorful, easy on the eyes… more appealing
Growing awareness	I was surprised by the fluctuations I have between days I could pay attention to what causes what, the chain of events. I could understand more what makes me feel bad I realized that I had a better day than I thought… the situation is not all negative… I found out that overall I am actually alright
More accurate report	The experience from the day is still fresh Was much easier. Sometimes during the interview [HRSD] I felt like I had to guess or bluff
**Weaknesses of HRSD-D**	
Lack of positive content	Depressed people sometimes have better days, even if just relatively. You need to have a way to let people express this… seeing only negative images, might even make them feel more depressed
**Key principles for the final HRSD-D**	
Functionality	Keep it user-friendly.@ It should be easy and simple as it is now
Personalization and customization of HRSD-D	Maybe you should let the participant choose one image for every item out of several options, then rate the image chosen.@ Something more rewarding at the end of answering instead of the banal "thank you for your response"… something more personal

#### Stability of symptom levels and fluctuations

We tested whether individual differences in average levels of MDD symptoms and in levels of daily fluctuations were stable features of the individual over the weeks. To this end, we divided the individual time series into quarters (weeks 1, 2, 3, and 4) and calculated individual means (iMs) and individual standard deviation (iSDs) for each week. We then correlated the resulting iM and iSD scores across each quarter to estimate the stability of these features. Results are presented in Tables 5 and 6 (respectively). A high correlation between weeks (> 0.6) suggests that individuals who showed low levels of symptoms or very little change over time in one week also showed low levels of symptoms or little change in the assessments of other weeks, respectively. Therefore, a high correlation reflects stability in the level of change over time. On average, levels of symptoms were highly stable over weeks, and levels of fluctuations displayed moderate stability rates from one week to the next. Thus, the observed individual differences in mean levels of MDD symptoms and in levels of fluctuations present different stable and meaningful patterns of symptomatology.

#### Predicting state items with trait corresponding items

Table 7 shows regression coefficient estimates and p values of the association between baseline trait scores (based on HRSD-DT), adjusted to age and gender, and corresponding daily state scores (based on HRSD-DS), using MLMs estimated by robust standard errors, and treating outcomes as continuously distributed. As shown, baseline trait scores were significant predictors of individual differences in state scores.

#### Qualitative feedback

We used thematic analysis of the transcripts. Because of their relatively short length (around 10 min on average), we followed the basic principles of inductive thematic analysis according to Braun and Clarke^51^: familiarizing ourselves with the data, generating initial codes, searching for themes, self-reviewing the themes, defining and naming the themes and producing the final report. Themes extracted from the semi-structured interviews were divided according to the three main topics of the interview: strengths of HRSD-DS, weaknesses of HRSD-DS, and key principles in developing the final tool. Themes are presented in Table 8.

## Stage 3: replication of stage 2

To test the replicability of the findings reported in stage 2, an independent preclinical sample was used.

### Method

Thirty-six participants took part in the replication stage. The procedure and the characteristics of the sample are reported in the online supplement.

### Results

The findings were largely replicated, including daily fluctuations of MDD symptoms, stability of symptoms levels and fluctuations, and predictions of state items based on corresponding trait items, as reported in stage 2. For further details see the online supplement. Most of the ICCs were slightly lower in the second sample, a decrease that was visible mostly for negative mood. Two items showed an increase in ICC; "loss of weight" and "suicidal thought."

## Discussion

The present study sought to develop the first digital image-based version of the HRSD, the HRSD-D, an innovative tool for MDD symptom monitoring. The final version of the HRSD-D includes the HRSD-DT, a one-time baseline report on general tendencies of the individual to experience MDD symptoms, and the HRSD-DS, a daily report on the experience of symptoms, capturing daily fluctuations of symptom severity. The findings demonstrate the high feasibility of daily monitoring using the HRSD-D, with 94% of participants completing all the study measurements. HRSD-D showed promising preliminary findings regarding validity and strong correlations with the original HRSD. HRSD-DS was found to be sensitive to daily fluctuations not captured by the weekly HRSD, and the findings were replicated in an independent sample. This study provides empirical evidence of the importance of exploring changes in depressive symptoms at a higher time resolution.

HRSD-D was also able to capture both differences between individuals in MDD symptoms (HRSD-DT) and daily fluctuations within individuals (HRSD-DS). The findings suggest a high level of stability of symptoms differentiating between individuals, which may serve as a trait-like characteristic of the individual, and a moderate level of fluctuations within individuals. This is indicated by highly stable levels of symptoms over weeks, and moderate stability in levels of fluctuations from one week to the next. On average, approximately half the variance in the daily manifestation of symptoms was found to be attributed to daily fluctuations within individuals. This is consistent with previous research on daily manifestations of mental symptoms^46^.

HRSD-D can provide an efficient, ecological, and fine-grained approach to research into the nature of MDD and may solve many of the drawbacks of traditional MDD symptom monitoring tools. First, HRSD-D provides more ecologically valid data and reduces reliance on memories. Weekly assessments might be inaccurate, as noted by our participants, and are especially prone to negative biases in MDD patients^52,53^. Second, HRSD-D is efficient and requires less time and resources than does HRSD. This is a significant advantage considering the poor motivation that often characterizes MDD patients^14,15^. From the point of view of researchers, HRSD-D provides an opportunity for assessment that does not require investing resources in the training of interviewers. Third, daily assessments provide a finer-grained clinical image than do weekly assessments^16^ and can support measurement-based care^54^. Daily monitoring is more sensitive to the dynamic pattern of symptoms and provides more precise information, which is especially beneficial given the finding that half the variability in MDD symptom manifestations is attributed to daily fluctuations. The rich data can be used to reveal correlational and causal links between symptoms to personalize treatment^16,55^. Fourth, HRSD-D may also deal with two main psychometric HRSD shortcomings^10^: the frequent daily measure might improve retest reliability, as short intervals between assessments were previously associated with much higher retest reliability scores^56^; and the uniform scaling turns the items into equal contributors to the global score. Finally, the two versions of HRSD-D make it possible to distinguish between the stable baseline features of symptomatology—general tendencies to experience MDD symptoms (a trait-like component) and the dynamic features — the daily manifestations of symptoms (a state-like component).

The ability of HRSD-D to separate assessment of trait- and state-like components may be essential to understanding the potential role of a stable level of depression vs. the development and progress of depression over time^17^. At any time, the level of depression is influenced by some constant trait and temporary changes (e.g., environmental stressors, social support, or biological dispositions^57^). This description of depression is consistent with our results showing that HRSD-DT scores explain half the variance of HRSD-DS. Additional support for the diverse roles of state and trait depression can be found in studies that showed that they correlate differently with psychopathology. For example, it was found that patients diagnosed with schizoaffective disorder show greater trait depressive symptoms than the healthy control group but not state depression^58^.

Evaluating trait depression is also important for clinical practice because it enables evaluating baseline depression without the noise originating from temporary changes. A limitation of measures not designed to measure trait depression is their inability to evaluate baseline levels of depression from which change related to treatment can be evaluated. Baseline assessment can be influenced by the state of the patient. For example, the baseline depression of patients being evaluated after a bad day at work or a fight at home may be higher than usual, and subsequent changes cannot be attributed to treatment. The high correlation that was found between average trait depression, as evaluated by the HRSD-DT, and trait depression evaluated by averaging the HRSD-DS suggest that HRSD-DT indeed measures consistent trait that is not influenced by temporary changes and therefore can accurately measure baseline depression. Finally, our sample reported both high anxiety and agitation, which may point to some overlap between the two. This overlap is consistent with previous literature indicating that these two items are loaded on the same factor^10^.

### Limitations and future directions

The limitations of this study can be divided into those of the current efforts to validate HRSD-D and those applicable to HRSD-D in general. The main limitation of the current study includes the use of a relatively small preclinical sample, which may affect the level of variability in some of the items (e.g., suicidal thoughts). But the variability in most of the items of the sample suggests that most of the items were sensitive enough to daily changes in symptoms even in a preclinical sample. Future studies should further explore the validity of HRSD-D with a larger sample of depressed patients. Another limitation is the fact that we measured symptoms daily, rather than with a time resolution where we did not expect symptoms to change (e.g., minutes). Therefore, we were not able to disentangle fluctuations within individuals and measurement errors. In this study, we followed the statistical pipeline suggested by Wright and Simms^46^ to test the utility of daily measurements, and Pollak et al.^31^ and Haim et al.^45^ in demonstrating the face validity of HRSD-D; future studies should complement the current findings with additional ones. Finally, although the present sample may represent the population it came from, future studies in different socio-cultural populations would be needed to further adjust the HRSD-D images, to both capture the content of the items of the original HRSD-D, and at the same time be culturally sensitive.

The limitations of HRSD-D itself have to do with the fact that it is based on self-report, and as such, on the desire of the participant to cooperate. Whereas the HRSD includes also the interviewer's viewpoint, HRSD-D is a pure self-report tool. A possible solution to this limitation may be the addition of implicit measures (e.g., audio recordings^59^) to HRSD-D. Another limitation, mentioned by our participants, is the possible overwhelming effect of negative content. This limitation emerged also in previous research on mental health apps^59^, pointing to the need for the inclusion of positive content.

## Conclusion

HRSD-D is an innovative image-based tool for MDD symptom monitoring, and to our knowledge, the first digital version of the gold standard HRSD. Our study demonstrates the feasibility of monitoring symptoms using HRSD-D and promising preliminary findings regarding the validity of the data collected. The development of two HRSD-D versions (HRSD-DT, HRSD-DS), assessing the trait-like and state-like components of symptomatology, enables researchers to explore each of them separately, as well as the important interactions between them.

## Supplementary Information

Supplementary Information.

## References

[1] World Health Organization. *Depression fact sheet, retrieved from*. https://www.who.int/en/news-room/fact-sheets/detail/depression (2020).

[2] Casacalenda N, Perry JC, Looper K (2002). Remission in major depressive disorder: a comparison of pharmacotherapy, psychotherapy, and control conditions. Am. J. Psychiatry.

[3] Rush AJ (2006). Acute and longer-term outcomes in depressed outpatients requiring one or several treatment steps: a STAR* D report. Am. J. Psychiatry.

[4] Beck, A. T., Steer, R. A. & Brown, G. K. *Manual for the Beck Depression Inventory-II*. vol. 1 (The Psychological Cooperation, 1996).

[5] Montgomery N, Åsberg H-A (1979). A new depression scale designed to be sensitive to change. Br J Psychiatry.

[6] Hamilton M (1960). A rating scale for depression. J. Neurol. Neurosurg. Psychiatry.

[7] Lam, R. W., Michalaak, E. E. & Swinson, R. P. *Assessment Scales in Depression, Mania and Anxiety*. (Taylor and Francis, 2006).

[8] Cusin, C., Yang, H., Yeung, A. & Fava, M. Rating scales for depression. *Handb. Clin. Rat. Scales Assess. Psychiatry Ment. Heal.* 7–35 (2009).

[9] Worboys M (2013). The Hamilton Rating Scale for Depression: The making of a “gold standard” and the unmaking of a chronic illness, 1960–1980. Chronic Illn..

[10] Bagby RM, Ryder AG, Schuller DR, Marshall MB (2004). The Hamilton depression rating scale: Has the gold standard become a lead weight?. Am. J. Psychiatry.

[11] Rohan KJ (2016). A protocol for the Hamilton Rating Scale for Depression: Item scoring rules, Rater training, and outcome accuracy with data on its application in a clinical trial. J. Affect. Disord..

[12] Mathews A, MacLeod C (2016). Cognitive vulnerability to emotional disorders. Annu. Rev. Clin. Psychol..

[13] Matt GE, Vázquez C, Campbell WK (1992). Mood-congruent recall of affectively toned stimuli: A meta-analytic review. Clin. Psychol. Rev..

[14] Treadway MT, Bossaller NA, Shelton RC, Zald DH (2012). Effort-based decision-making in major depressive disorder: a translational model of motivational anhedonia. J. Abnorm. Psychol..

[15] Smith B (2013). Depression and motivation. Phenomenol. Cogn. Sci..

[16] Fisher AJ (2015). Toward a dynamic model of psychological assessment: Implications for personalized care. J. Consult. Clin. Psychol..

[17] Zilcha-Mano S (2020). Toward personalized psychotherapy: The importance of the trait-like distinction for understanding therapeutic change. Am. Psychol..

[18] Zilcha-Mano S (2019). Major developments in methods addressing for whom psychotherapy may work and why. Psychother. Res..

[19] Myin-Germeys I (2009). Experience sampling research in psychopathology: opening the black box of daily life. Psychol. Med..

[20] Myin-Germeys I (2018). Experience sampling methodology in mental health research: new insights and technical developments. World Psychiatry.

[21] Telford C, McCarthy-Jones S, Corcoran R, Rowse G (2012). Experience sampling methodology studies of depression: the state of the art. Psychol. Med..

[22] Napa Scollon, C., Prieto, C.-K. & Diener, E. Experience sampling: Promises and pitfalls, strength and weaknesses. in *Assessing Well-Being* 157–180 (Springer, 2009).

[23] Verhagen SJW, Hasmi L, Drukker M, van Os J, Delespaul PAEG (2016). Use of the experience sampling method in the context of clinical trials. Evid. Based. Ment. Health.

[24] Ebner-Priemer UW, Trull TJ (2009). Ecological momentary assessment of mood disorders and mood dysregulation. Psychol. Assess..

[25] Miller G (2012). The smartphone psychology manifesto. Perspect. Psychol. Sci..

[26] Van Berkel N, Ferreira D, Kostakos V (2017). The experience sampling method on mobile devices. ACM Comput. Surv..

[27] Sim I (2019). Mobile devices and health. N. Engl. J. Med..

[28] Torous J, Friedman R, Keshavan M (2014). Smartphone ownership and interest in mobile applications to monitor symptoms of mental health conditions. JMIR mHealth uHealth.

[29] Yang, L. *et al.* Your activities of daily living (YADL): An image-based survey technique for patients with arthritis. *arXiv Prepr.* https://arxiv.org/abs/1601.03278 (2016).

[30] Meschtscherjakov A, Weiss A, Scherndl T (2009). Utilizing emoticons on mobile devices within ESM studies to measure emotions in the field. Proc. MME Conjunct. MobileHCI.

[31] Pollak, J. P., Adams, P. & Gay, G. PAM: a photographic affect meter for frequent, in situ measurement of affect. in *Proceedings of the SIGCHI Conference on Human Factors in Computing Systems* 725–734 (2011).

[32] Sobolev M (2021). The Digital Marshmallow Test (DMT) diagnostic and monitoring mobile health app for impulsive behavior: development and validation study. JMIR mHealth uHealth.

[33] Desmet PMA, Vastenburg MH, Romero N (2016). Mood measurement with Pick-A-Mood: review of current methods and design of a pictorial self-report scale. J. Des. Res..

[34] Trull, T. J. & Ebner-Priemer, U. W. Using experience sampling methods/ecological momentary assessment (ESM/EMA) in clinical assessment and clinical research: introduction to the special section. (2009). 10.1037/a0017653 PMC4255457 19947780

[35] Bauer S, Moessner M (2012). Technology-enhanced monitoring in psychotherapy and e-mental health. J. Ment. Heal..

[36] Van Ameringen M, Turna J, Khalesi Z, Pullia K, Patterson B (2017). There is an app for that! The current state of mobile applications (apps) for DSM-5 obsessive-compulsive disorder, posttraumatic stress disorder, anxiety and mood disorders. Depress. Anxiety.

[37] Lui JHL, Marcus DK, Barry CT (2017). Evidence-based apps? A review of mental health mobile applications in a psychotherapy context. Prof. Psychol. Res. Pract..

[38] Torous J (2015). Utilizing a personal smartphone custom app to assess the patient health questionnaire-9 (PHQ-9) depressive symptoms in patients with major depressive disorder. JMIR Ment. Heal..

[39] Chung K (2018). Development and evaluation of a mobile-optimized daily self-rating depression screening app: A preliminary study. PLoS ONE.

[40] Manassis K (2009). Mood assessment via animated characters: A novel instrument to evaluate feelings in young children with anxiety disorders. J. Clin. Child Adolesc. Psychol..

[41] Laurans, G. F. G. & Desmet, P. M. A. Introducing PREMO2: New directions for the non-verbal measurement of emotion in design. in *Out of Control: Proceedings of the 8th International Conference on Design and Emotion* 11–14 (2012).

[42] Broekens J, Brinkman W-P (2013). Affect Button: A method for reliable and valid affective self-report. Int. J. Hum. Comput. Stud..

[43] Spielberger, C. D., Gorsuch, R. L., Lushene, R., Vagg, P. R. & Jacobs, G. A. Manual for the STATE-TRAIT ANXIETY INVENtory (Form Y) Mind Garden. *Palo Alto, CA* (1983).

[44] Carmody TJ (2006). The Montgomery Äsberg and the Hamilton ratings of depression: a comparison of measures. Eur. Neuropsychopharmacol..

[45] Haim, S. *et al.* The mobile photographic stress meter (MPSM) a new way to measure stress using images. in *Adjunct Proceedings of the 2015 ACM International Joint Conference on Pervasive and Ubiquitous Computing and Proceedings of the 2015 ACM International Symposium on Wearable Computers* 733–742 (2015).

[46] Wright AGC, Simms LJ (2016). Stability and fluctuation of personality disorder features in daily life. J. Abnorm. Psychol..

[47] Kitchenham B, Pfleeger SL (2002). Principles of survey research: part 5: populations and samples. ACM SIGSOFT Softw. Eng. Notes.

[48] Connelly LM (2008). Pilot studies. Medsurg Nurs..

[49] Goodwin J, Cummins J, Behan L, O’Brien SM (2016). Development of a mental health smartphone app: Perspectives of mental health service users. J. Ment. Heal..

[50] Littell, R. C., Milliken, G. A., Stroup, W. W., Wolfinger, R. D. & Oliver, S. *SAS for Mixed Models*. (SAS publishing, 2006).

[51] Braun V, Clarke V (2006). Using thematic analysis in psychology. Qual. Res. Psychol..

[52] Clark DM, Teasdale JD (1982). Diurnal variation in clinical depression and accessibility of memories of positive and negative experiences. J. Abnorm. Psychol..

[53] Greenberg MS, Beck AT (1989). Depression versus anxiety: a test of the content-specificity hypothesis. J. Abnorm. Psychol..

[54] Scott K, Lewis CC (2015). Using measurement-based care to enhance any treatment. Cogn. Behav. Pract..

[55] Fisher AJ, Boswell JF (2016). Enhancing the personalization of psychotherapy with dynamic assessment and modeling. Assessment.

[56] Trajković G (2011). Reliability of the hamilton rating scale for depression: A meta-analysis over a period of 49 years. Psychiatry Res..

[57] Teasdale JD (1988). Cognitive vulnerability to persistent depression. Cogn. Emot..

[58] Chiappelli J, Nugent KL, Thangavelu K, Searcy K, Hong LE (2014). Assessment of trait and state aspects of depression in schizophrenia. Schizophr. Bull..

[59] Rickard N, Arjmand H-A, Bakker D, Seabrook E (2016). Development of a mobile phone app to support self-monitoring of emotional well-being: a mental health digital innovation. JMIR Ment. Heal..

