# Supplementary material for: Development and evaluation of the HRSD-D, an image-based digital measure of the Hamilton rating scale for depression
Source: Sci Rep. 2022 Aug 22;12:14342. doi: 10.1038/s41598-022-18434-y (PMC9395406; doi:10.1038/s41598-022-18434-y)
Supplement: Supplementary file 1 — Supplementary Information. [file 41598_2022_18434_MOESM1_ESM.pdf]

Stage 3 Method and Results

Method

Participants

Thirty-six first-year undergraduate students at the university of Haifa, participated in the replication stage of the current study. Recruitment was based on non-probabilistic convenience sampling which are common in pilot studies (Kitchenham & Pfleeger, 2002; Connelly, 2008). Table S1 presents the demographic and clinical characteristics of the sample.

Procedure

The procedure was identical to the one reported for the original sample, excluding the weekly interviews of the original HRSD

Measures

HRSD State-Like (HRSD-DS). HRSD-DS was used as reported in the original sample.

HRSD Trait-Like (HRSD-DT). HRSD-DT was used as reported in the original sample**.**

Statistical Analyses

Daily Fluctuations in MDD Symptoms. Examination of the ability of HRSD-DS to capture the daily fluctuations in MDD symptoms was identical to the one reported for the original sample.

Stability of Symptom Levels and Fluctuations. Examination of the ability of HRSD-DS to capture the stable features of individual symptomatology and ascertain whether individuals maintain their relative position to each other in their level and variance, was identical to the one reported for the original sample.

Predicting State Items Based on Corresponding Trait Items. Examination of the relation between trait-like and state-like scores was identical to the one reported for the original sample.

Results

Adherence rate for HRSD-DS stands on 97.3% over the 28-day study period.

Daily Fluctuations in MDD Symptoms

We examined the proportion of variance in daily HRSD-DS scores attributable to between-persons differences by calculating ICCs from intercept only MLMs. ICCs for HRSD-DS items are shown in Table 3

(note that in Tables 3-6 the names of the items are listed according to the titles displayed in HRSD, not necessarily as they appear in the original HRSD). All ICCs were significant ($p < .001$). At the item level, the average ICC was .42 (range: .21–.68). This suggests that, on average, approximately 60% the variance in the daily manifestation of MDD symptoms can be attributed to individual differences, and the remaining 40% to daily fluctuations. At the same time, we found differences depending on the individual item. The items concerning depressed mood, feelings of guilt, low motivation, somatic symptoms of anxiety, loss of appetite and low sexual desire had the lowest ICCs, indicating that most of the variance in their manifestations was due to daily fluctuations. Loss of weight was associated with the largest ICCs, indicating that most of the variance in their manifestations was due to stable individual differences, rather than daily fluctuations. Table 3 also summarizes patterns of endorsement for each HRSD-DS item. The third column of the table shows that the items varied considerably in the proportion of the sample that endorsed them, ranging from 71% of the sample that endorsed agitation and anxiety, to only 7% of the sample that endorsed suicidal thoughts, and 30% that endorsed loss of appetite, low energy and loss of weight.

Stability of Symptom Levels and Fluctuations

We tested whether individual differences in average levels of MDD symptoms and in levels of daily fluctuations were stable features of the individual over the weeks. To this end, we divided the individual time series into quarters (weeks 1, 2, 3, and 4) and calculated individual means (iMs) and individual standard deviation (iSDs) for each week. We then correlated resulting iM and iSD scores across each quarter to estimate the stability of these features. Results are presented in Table 4a, and Table 4b (respectively). Stability is obtained if there is a high correlation (> .6) between weeks. On average, levels of symptoms were highly stable over weeks, and levels of fluctuations displayed moderate stability rates from one week to the next. Thus, the observed individual differences in mean levels of MDD symptoms and in levels of fluctuations present different stable and meaningful patterns of symptomatology.

Predicting State Items with Trait Corresponding Items

Table 5 shows regression coefficient estimates and p values of the association between baseline trait scores (based on HRSD-DT), adjusted to age and gender, and corresponding daily state scores (based on

HRSD-DS), using MLMs estimated by robust standard errors, and treating outcomes as continuously distributed. As shown, baseline trait scores were significant predictors of individual differences in state scores in all items except suicidal thoughts. Additionally, HRSD-DT scores correlated positively and significantly-with the monthly average of HRSD-DS scores (r =.70, $p<.001$).

Table S2

Demographic and Clinical Characteristics of the Replication Sample

	n	%
Gender		
Female	25	67.6
Male	10	27
Marital status		
Single	19	51.4
Married/partnered	16	43.2
Divorced/widowed	0	0
Highest educational level		
High school/some college	25	67.6
Graduate college	10	27
Graduate degree	0	0
Diagnosed in the past		
Major Depressive Disorder	2	5.4
Dysthymia	0	0
Other	3	8.1
Currently in treatment		
Psychotherapy	9	24.3
Medications	0	0

Note. N = 35. Participants were on average 22.47 years old (SD = 3.04). One participant did not complete the demographic section.

Table S3

Descriptive Statistics for Endorsement of Daily Manifestations of MDD Symptoms Based on HRSD-DS

Item	Estimated coefficient of reliability, ICC (95% CI)	Percentage endorsement					
		Ever	Daily				
		>1	1	2	3	4	5
Depressed mood	0.21 (0.13, 0.31)	56	44	25	16	9	6
Feelings of guilt	0.37 (0.26, 0.49)	50	50	23	13	8	6
Suicidal thoughts	0.46 (0.34, 0.58)	7	93	3	2	1	1
Difficulties falling asleep	0.44 (0.32, 0.56)	47	53	13	13	10	11
Restless sleep	0.40 (0.29, 0.52)	44	56	13	11	9	10
Early spontaneous awakening	0.44 (0.33, 0.56)	40	60	11	11	10	8
Low motivation (work/activities)	0.32 (0.22, 0.43)	64	36	24	23	9	8
Agitation	0.43 (0.31, 0.55)	71	29	17	22	19	13
Anxiety	0.43 (0.32, 0.55)	71	29	21	22	16	13
Somatic symptoms of anxiety	0.38 (0.27, 0.50)	52	48	19	16	9	7
Loss of appetite	0.34 (0.23, 0.46)	30	70	13	9	4	3
Low energy	0.42 (0.31, 0.55)	30	35	22	19	13	11
Low sexual desire	0.36 (0.25, 0.48)	39	61	14	15	6	4
Hypochondriasis	0.44 (0.33, 0.56)	37	63	16	12	5	3
Loss of weight	0.68 (0.57, 0.78)	30	70	11	8	6	6
Total	0.55 (0.43, 0.67)	100					

Note. Person-level *N* =36; daily-level *N* =981; ICC = intraclass correlation; CI = confidence interval.

Table S4a

Stability in Individual Level of Symptoms (Mean)over 4 weeks of the Assessment Period

Daily item	r 1-2	r 1-3	r 1-4	r 2-3	r 2-4	r 3-4	mean	min	max
	Mean level (iM)								
Depressed mood	0.28	0.44	0.22	0.70	0.45	0.49	0.43	0.22	0.70
Feelings of guilt	0.67	0.72	0.43	0.85	0.54	0.73	0.66	0.43	0.85
Suicidal thoughts	0.79	0.77	0.62	0.92	0.83	0.91	0.81	0.62	0.92
Difficulties falling asleep	0.73	0.68	0.55	0.80	0.63	0.72	0.69	0.55	0.80
Restless sleep	0.70	0.67	0.53	0.91	0.58	0.66	0.68	0.53	0.91
Early spontaneous awakening	0.72	0.51	0.61	0.82	0.77	0.70	0.69	0.51	0.82
Low motivation (work/activities)	0.67	0.73	0.35	0.81	0.59	0.50	0.61	0.35	0.81
Agitation	0.83	0.75	0.73	0.81	0.71	0.82	0.78	0.71	0.83
Anxiety	0.79	0.61	0.63	0.71	0.68	0.82	0.71	0.61	0.82
Somatic symptoms of anxiety	0.70	0.50	0.51	0.83	0.79	0.67	0.67	0.50	0.83
Loss of appetite	0.66	0.72	0.66	0.80	0.63	0.71	0.70	0.63	0.80
Low energy	0.74	0.77	0.52	0.92	0.72	0.68	0.73	0.52	0.92
Low sexual desire	0.52	0.53	0.57	0.78	0.76	0.80	0.66	0.52	0.80
Hypochondriasis	0.70	0.65	0.48	0.87	0.76	0.73	0.70	0.48	0.87
Loss of weight	0.86	0.89	0.88	0.91	0.86	0.95	0.89	0.86	0.95
Items r's Mean	0.69	0.66	0.55	0.83	0.69	0.73			
Items r's Min	0.28	0.44	0.22	0.70	0.45	0.49			
Items r's Max	0.86	0.89	0.88	0.92	0.86	0.95			

Note. N=36. All correlations greater than 0.33 are significant at p <.05.

Table S4b

Stability in Individual Levels of Fluctuations (SD) over 4 weeks of the Assessment Period

	Variability (iSD)						mean
Depressed mood	0.34	0.40	0.29	0.49	0.24	0.46	0.37
Feelings of guilt	0.40	0.46	0.50	0.71	0.57	0.62	0.54
Suicidal thoughts	0.29	0.30	-0.01	0.39	0.22	0.61	0.30
Difficulties falling asleep	0.40	0.32	0.18	0.43	0.31	0.48	0.35
Restless sleep	0.11	0.28	0.35	0.42	0.37	0.72	0.38
Early spontaneous awakening	0.41	0.23	0.29	0.35	0.31	0.30	0.32
Low motivation (work/activities)	0.34	0.50	0.36	0.54	0.68	0.45	0.48
Agitation	0.34	0.25	0.34	0.53	0.57	0.52	0.43
Anxiety	0.27	0.15	0.43	0.44	0.38	0.52	0.37
Somatic symptoms of anxiety	0.49	0.37	0.57	0.50	0.46	0.42	0.47
Loss of appetite	0.49	0.52	0.42	0.66	0.75	0.65	0.58
Low energy	0.48	0.46	0.52	0.59	0.54	0.68	0.55
Low sexual desire	0.75	0.49	0.48	0.79	0.69	0.82	0.67
Hypochondriasis	0.32	0.33	0.21	0.47	0.44	0.59	0.39
Loss of weight	0.52	0.57	0.61	0.73	0.73	0.81	0.66
Mean r's items	0.40	0.38	0.37	0.54	0.48	0.58	0.46

Note. N = 36. All correlations greater than 0.33 are significant at p <.05.

Table S5

Predicting Individual Differences in Rates of Daily state items from their corresponding baseline traits

Item	Estimate	SE	Partial η^2	p
Depressed mood	0.24	0.05	0.03	<.0001
Feelings of guilt	0.40	0.03	0.20	<.0001
Suicidal thoughts	0.02	0.03	0.00	0.4229
Difficulties falling asleep	0.65	0.03	0.34	<.0001
Restless sleep	0.42	0.03	0.15	<.0001
Early spontaneous awakening	0.44	0.04	0.13	<.0001
Low motivation (work/activities)	0.20	0.04	0.03	<.0001
Agitation	0.41	0.04	0.11	<.0001
Anxiety	0.47	0.04	0.13	<.0001
Somatic symptoms of anxiety	0.31	0.04	0.07	<.0001
Loss of appetite	0.37	0.04	0.07	<.0001
Low energy	0.49	0.04	0.13	<.0001
Low sexual desire	0.60	0.05	0.13	<.0001
Hypochondriasis	0.27	0.03	0.08	<.0001
Loss of weight	0.54	0.03	0.28	<.0001
Total	0.59	0.03	0.26	<.0001

Note. Person-level *N* =27; daily-level *N*=734. All models estimated controlling for gender and age. partial η^2: represent an estimate of how much variance in the state item is accounted for by the trait item. According to Cohen, J. (1988) 0.01 partial η^2 effect considered as small, 0.06 considered as medium and 0.14 considered as large. Nine participants did not complete the HRSD-DT due to technical problems.

Additional Characteristics of the Original Sample

Table S6

Clinical Characteristics of the Original Sample

	n	%
Level of depression at baseline[a]		
Not depressed	14	28.6
Subthreshold	10	20.4
Mild	17	34.7
Moderate	6	12.2
Severe	2	4.1
Diagnosed in the past		
Major Depressive Disorder	8	16
Dysthymia	1	2
Other	8	16
Currently in treatment		
Psychotherapy	10	20
Medications	2	4

Note. *N* = 50. Levels of depression are in line with the National Collaborating Centre for Mental Health (UK; 2010).
